# Positivity to p-ANCA in patients with status epilepticus

**DOI:** 10.1186/1471-2377-14-148

**Published:** 2014-07-17

**Authors:** Edoardo Ferlazzo, Antonio Gambardella, Marina Bellavia, Sara Gasparini, Laura Mumoli, Angelo Labate, Vittoria Cianci, Concetta Russo, Umberto Aguglia

**Affiliations:** 1 Department of Medical and Surgical Sciences, Magna Graecia University, Catanzaro, Italy; 2 Regional Epilepsy Centre, Bianchi-Melacrino-Morelli Hospital, Reggio Calabria, Italy; 3 Institute of Neurology, University Magna Graecia - Catanzaro, Italy

**Keywords:** Vasculitis, Seizures, p-ANCA, Epilepsy, Status epilepticus, Death

## Abstract

**Background:**

Status epilepticus (SE) may occur in the setting of several internal or neurologic diseases. Anti-neutrophilic cytoplasmic antibodies (ANCA) are a group of Ig that may be observed in patients with different autoimmune disorders but are particularly associated with systemic vasculitis named ANCA-associated-vasculities (AAV). We herein report 3 patients with SE and positivity to p-ANCA.

**Case presentation:**

One patient had a catastrophic evolution and died 5 months after disease onset. The other two patients had a good outcome and remained seizure-free at 30 months and 5 years of follow-up respectively.

**Conclusion:**

This report highlights the importance of considering ANCA dosage in patients with SE of unclear origin.

## Background

Status epilepticus (SE) may occur in the setting of several neurologic or internal diseases. Anti-neutrophilic cytoplasmic antibodies (ANCA) are a group of Ig that may be observed in patients with different autoimmune disorders but are particularly associated with systemic vasculitis. ANCA-associated vasculitis (AAV) are rare systemic autoimmune diseases affecting small to medium sized blood vessels [1]. AAV include granulomatosis with polyangiitis (formerly Wegener’s granulomatosis), microscopic polyangiitis and eosinophilic granulomatosis with polyangiitis (formerly Churg-Strauss syndrome). These conditions may rapidly lead to multiple organ failure and death if not early diagnosed [1]. AAV may involve different organs especially upper and lower respiratory tracts, kidneys, skin. Neurological manifestations are observed in about 20% of patients, in particular peripheral neuropathy [1]. Seizures, headache and stroke have rarely been reported in AAV [2-7]. We herein report 3 patients presenting SE of unclear origin, whose sera were positive for p-ANCA.

## Cases presentation

### Patient 1

**Figure 1:**
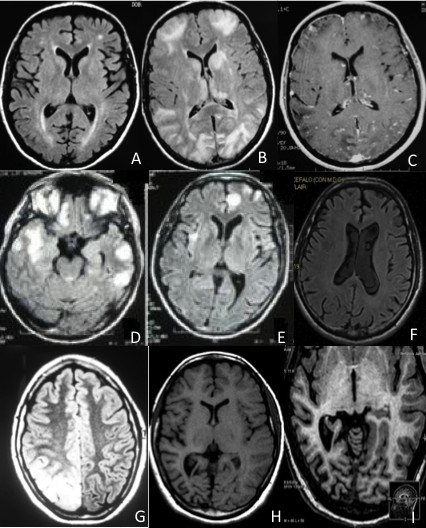
**Brain MRI in our series. A** (patient 1, at entry): hyperintense spots in axial FLAIR sequence involving periventicular and subcortical white matter. **B**, **C** (patient 1, one month later): axial FLAIR sequence showing multiple cortico-subcortical hyperintense lesions, some of which enhanced after gadolinium administration (axial T1: C). No meningeal enhancement is observed. **D**, **E** (patient 2, at entry): multiple nodular cortico-subcortical FLAIR hyperintense lesions involving fronto-temporal and insular regions. **F** (patient 2, three years later): axial FLAIR sequence revealing no abnormality. **G** (patient 3, three years after disease onset): axial FLAIR sequence showing a vast hyperintense cortico-subcortical temporo-occipital lesion. **H**, **I** (patient 3, two years after treatment onset with azathioprine): axial T1 revealing mild cortico-subcortical atrophy involving right temporal and occipital lobes with dilatation of posterior horn of right lateral ventricle. Magnification of right posterior atrophy by axial IR (I).

A 48-year-old man was diagnosed AAV with extracapillary necrotizing glomerulonephritis confirmed by laboratory analyses and kidney biopsy. He was treated with a single iv bolus of cyclophosphamide 5 mg and iv methylprednisolone 250 mg/day for 3 days, followed by oral prednisone 1 mg/kg/day. Two months later he was admitted because of generalized convulsive SE treated with iv bolus of lorazepam 4 mg. Post-ictal neurological examination showed no deficits. Neither skin lesions nor involvement of joints were evident. Brain MRI (Figure 1A) showed small-sized hyperintense T2- and FLAIR-weighted lesions in periventricular and subcortical white matter without contrast enhancement. A vast haematological screening was normal and included haemachrome, liver function, electrolytes, coagulation, angiotensin-converting enzyme, microbiological tests for hepatitis, toxoplasma, rubella, CMV, HSV 1 and 2, syphilis, ferritin, immunoglobulin dosage, antinuclear antibodies (ANA), extractable nuclear antibody (ENA) including anti-dsDNA, anti-Ro/SSA, anti-La/SSB, anti-histone, anti-nRNP, anti-Jo-1, anti-Scl70, anti-Sm, c-ANCA, rheumatoid factor, C3, C4, lupus anticoagulant (LAC), anti-thyroid antibodies, alpha-fetoprotein, carcino-embryonic antigen, neuronal-specific enolase, prostatic-specific antigen, CA19-9. Abnormal results were serum creatinine 7.82 mg/dl (not worsened as compared to preadmission value), ESR 57 mm/h, C reactive protein 102 mg/l, positivity to p-ANCA (as detected by both indirect immunofluorescence and ELISA for ANCA-MPO), microhaematuria. CSF analysis, including cultures, was negative. Chest CT scan revealed interstitial lung disease. Spirometry showed mild restrictive ventilatory defect. Electrocardiography, echocardiography and abdominal MRI were normal. He was treated with iv methylprednisolone 1 gr /day for 5 days and was discharged with carbamazepine 400 mg/day and prednisone 1 mg/kg/day. One month later, he was again admitted because of sudden confusional state with hematemesis, haemoptysis, nosebleed and macroscopic haematuria. Blood pressure was normal. Haematological examination showed severe anaemia and multiple organ (kidney, pancreas, liver) failure. CSF analysis revealed high protein level (417 md/dl; n.v. 18–43). Polygraphic recording revealed asterixis, slow background activity and bilateral triphasic waves. Brain MRI showed multiple cortico-subcortical lesions, some of which enhanced after gadolinium administration (Figure 1B, C). Patient died for haemorrhagic shock 3 days after admission, 5 months after disease onset.

### Patient 2

A 44-year-old coeliac man presented with fever, lymphadenopathy and haemolytic anaemia of unknown origin, treated with prednisone 1 mg/kg/day. In the same epoch he presented weekly short-lasting episodes of epigastric aura with sweating and was prescribed levetiracetam 1 gr/day. Two months later he was admitted for refractory SE with left hemibody clonic jerks not responding to i.v. boli of lorazepam 4 mg and phenytoin 20 mg/kg. Neurological examination was otherwise normal. He was given oral levetiracetam 2 gr/day and topiramate 200 mg/day, but clonic jerks persisted. EEG showed periodic lateralized epileptic activity over right fronto-centro-temporal regions. Neither skin lesions nor joints involvement joints were evident. Brain MRI showed multiple lesions involving cortico-subcortical fronto-temporal regions (Figure 1D, E) without contrast enhancement. The same laboratory panel as patient 1 was unremarkable except for ANA 1:320 (speckled pattern as detected by indirect fluorescence antibody test), ESR 24 mm/h, C reactive protein 257 mg/l, positivity to p-ANCA (as detected by both indirect immunofluorescence and ELISA for ANCA-MPO). Urinalyses, including microalbuminuria, and serum creatinine were normal. CSF analysis showed oligoclonal bands; cultures were negative. Abdominal and pelvic CT scans, electrocardiography and echocardiography were normal. Methylprednisolone 1 gr/day was administered for 5 days. He was then given i.v. rituximab (600 mg/week for 4 weeks). During the following 5-year follow-up he had no relapses and remained seizure-free with levetiracetam 2 gr/day. A control MRI performed 3 years later was normal (Figure 1F).

### Patient 3

**Figure 2:**
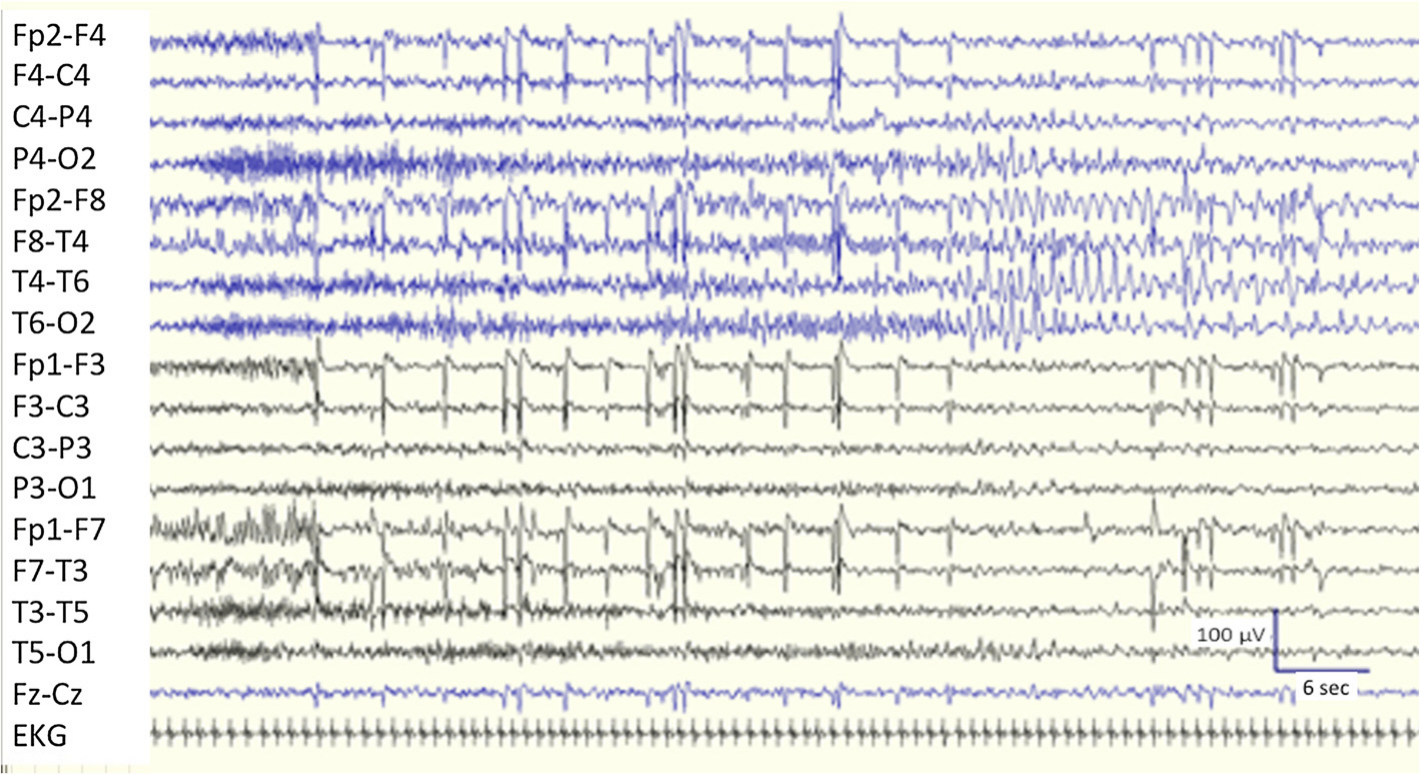
**EEG showing recruiting, fast “tonic” activity over right posterior head regions, predominant over right occipital lead (O2), followed by sharp theta activity over ipsilateral temporo-occipital leads.** Seizure lasts about 75 seconds (bipolar montage, scalp electrodes placed according to the International 10–20 system). The trace is compressed to show seizure in its all duration.

A previously healthy 17-year-old girl started presenting long-lasting episodes of vision of colored circles, followed by headache. At age 18 she developed almost continuous short-lasting episodes of sensation that “eyes move like windscreen wiper”. A brain MRI performed at that time revealed a vast hyperintense lesion in T2, FLAIR and diffusion-weight images, involving the right temporo-occipital lobes. Muscle biopsy was normal and genetic test to detect MELAS mtDNA mutations was unremarkable. Molecular screening (direct sequence analysis and multiplex ligation-dependent probe amplification) of DNA polymerase subunit gamma-1 (POLG1) was normal. Treatment with topiramate 200 mg/day was started and she remained asymptomatic over 2 years. Thereafter, topiramate had to be discontinued due to side effects and carbamazepine up to 800 mg/day was given. Several brain MRI performed 6, 12 and 24 months later were normal. Afterwards, she was admitted for blurred vision and frequent short-lasting sensations that “eyes move like windscreen wiper”. EEG (Figure 2) disclosed recurrent occipital seizures every 3–10 minutes clinically characterized by horizontal nystagmus towards the left. Neurological examination revealed left lower homonymous quadrantanopia. SE stopped after i.v. bolus of diazepam 10 mg. Neither skin lesions nor involvement of joints were evident. Brain MRI showed a well-defined right temporo-occipital lesion (Figure 1G) without contrast enhancement. The same haematological screening as patients 1 and 2, revealed ANA 1:320 (speckled pattern as detected by indirect fluorescence antibody test) and positivity to p-ANCA (as detected by both indirect immunofluorescence and ELISA for both ANCA-MPO and ANCA-PR3). Moreover, C reactive protein and the erythrocyte sedimentation rate were elevated. Tests for coeliac disease, syphilis, Lyme disease, anti-glutamate receptor (2 and 3) antibodies and paraneoplastic antibodies were negative. CSF analysis, including cultures, was normal. Urinalyses, including microalbuminuria, were normal. Electroneuromyography, electrocardiography, chest X-ray, abdominal and pelvic ultrasound were unremarkable. She was given carbamazepine 800 mg/day. In the following 14 months, two similar episodes of occipital SE associated with transitory MRI abnormalities recurred and resolved with appropriate antiepileptic therapy. After the last SE, brain MRI revealed right temporo-parieto-occipital atrophy (Figures 1H, I). Due to the persistence of positive p-ANCA and ANA, she was given carbamazepine 800 mg/day, oral prednisone 50 mg/day for one month, followed by azathioprine 75 mg/day, remaining seizure-free during 30-month follow-up. The right temporo-parietal-occipital atrophy remained unchanged in control MRI.

## Conclusions

In our series, SE represented the main or first neurological manifestation. Although the presence of a CNS vasculitis was not confirmed by brain biopsy or angiography, positivity to p-ANCA prompted us to suspect an autoimmune aetiology and to begin immunotherapy. Moreover, the proposed diagnostic criteria of AAV are based on systemic symptoms and signs, serological, radiological or histological findings, exclusion of other conditions as malignancy, infections, drugs, sarcoidosis, secondary vasculitis [8]. Patient #1 fulfilled the diagnostic criteria for AAV [8] with central nervous system (CNS) involvement. Indeed, he had positivity for p-ANCA along with systemic signs of disease (glomerulonephritis and pulmonary interstitiopathy), histological proof of vasculitis and no other diagnosis accounting for the clinical picture. MRI picture could suggest a “posterior reversible encephalopathy syndrome or PRES [9]. However, contrast enhancement at MRI, normality of blood pressure, and absence of worsening of kidney function during SE, does not support this hypothesis. Patient 2 also had positivity of p-ANCA, in the presence of non-specific signs of vasculitis (fever, lymphadenopathy and haemolytic anaemia) along with absence of other diagnosis accounting for the clinical picture, thus fulfilling only 2 out of 3 necessary criteria. However, the positivity of p-ANCA prompted us to start treatment with rituximab with good outcome. Patient 3 also fulfilled 2 out of 3 proposed criteria for AAV (positivity for p-ANCA and exclusion for other conditions accounting for her symptoms, including the above-mentioned diseases). An atypical form of Rasmussen encephalitis could be suggested in this patient [10]. However, the clinical findings as well as the extensive laboratory investigation, including the MRI features (with no characteristic evolutional changes of MRI occurring at long-term follow-up, including the atrophy of the head of the caudate nucleus) made this diagnosis very unlikely. Therefore, all these data, along with the prompt response to azathioprine treatment, strongly suggested the diagnosis of an autoimmune disorder with p-ANCA positivity limited to CNS in this young patient. Of interest, in the same patient, we also excluded genetic conditions potentially leading to focal brain lesions and seizure disorder, including POLG mutations. Indeed, it is known that POLG mutations may cause a progressive neurological disorder usually starting in teens with occipital lobe epilepsy and frequent SE [11]. This observation suggests that AAV should be considered in the differential diagnosis with POLG mutations in patients presenting with occipital seizures or SE.

A study [12] on natural history of AAV found that average patient survival is about five months and more than 90% of patients die within two years. Standard treatment of AAV usually consists of inducing remissions with high dose steroids or cyclophosphamide, and maintaining remission with immunosuppressants. New therapeutic agents include rituximab, a monoclonal antibody depleting B cell, approved by FDA in US for use in AAV. The striking and long-lasting response in patient 2 confirms that rituximab may represent a therapeutic alternative to classic immunosuppressant for AAV in some patients. Patient 3 too had a good outcome and remained seizure-free at follow-up with immunosuppressant and antiepileptic treatment. On the contrary, patient 1 had extremely rapid and fatal evolution due to small vessel necrotizing vasculitis and multiple organs bleeding despite treatment with steroids.

The exact nature of cortico-subcortical hyperintensities associated with SE remains elusive, even if such changes may be at least in part related to cytotoxic edema [13]. Regardless the pathophysiological mechanism, we believe that the present series strengthens the view that both SE and brain hyperintensities should be targets of rational treatment, as there is now agreement that SE and its combination with sterile brain inflammation are the major determinants of sequelae [14]. In this way, it may be questioned if an earlier immunosuppressive treatment in our patient #3 could have prevented the irreversible loss of brain parenchyma.

In conclusion, physicians should consider ANCA dosage in patients presenting with otherwise unexplained SE or seizures, inflammatory changes at brain MRI, with or without other systemic signs or symptoms of AAV. Indeed, in this peculiar clinical setting, ANCA positivity may lead physicians to suspect an autoimmune aetiology and to promptly start adequate immunotherapy.

### Consent

Written informed consents were obtained from the patients #2,3 for publication of this case series and any accompanying images. Written informed consent was obtained from the wife of patient 1 for publication of this case series and any accompanying images. A copy of the written consent is available for review by the Editor-in-Chief of this journal.

## Competing interests

All authors declare that they have no competing interests. There is no funding or financial support.

## Authors’ contributions

EF: drafting/revising/critique the manuscript, study concept or design, collection of data. AG: revising/critique the manuscript, study concept or design, collection of data, supervision. MB: revising/critique the manuscript. SG: revising/critique the manuscript. LM: revising/critique the manuscript, collection of data. AL: revising/critique the manuscript, collection of data. VC: revising/critique the manuscript, collection of data. CR: revising/critique the manuscript, study concept or design, collection of data. UA: drafting/revising/critique the manuscript, study concept or design, collection of data. All authors read and approved the final manuscript.

## Pre-publication history

The pre-publication history for this paper can be accessed here:

http://www.biomedcentral.com/1471-2377/14/148/prepub
